# Efficacy of fluralaner against *Otodectes cynotis* infestations in dogs and cats

**DOI:** 10.1186/s13071-016-1954-y

**Published:** 2017-01-16

**Authors:** Janina Taenzler, Christa de Vos, Rainer K. A. Roepke, Régis Frénais, Anja R. Heckeroth

**Affiliations:** 1 MSD Animal Health Innovation GmbH, Zur Propstei, 55270 Schwabenheim, Germany; 2 Clinvet International Pty (Ltd), Uitsigweg, Bainsvlei, 9338 Bloemfontein, Free State South Africa; 3 MSD Animal Health Innovation SAS, Rue O. de Serres, 49071 Beaucouzé, Cedex France

**Keywords:** Bravecto™, Bravecto™ spot-on solution, Cat, Chewable tablets, Dog, Ear mite, Efficacy, Fluralaner, Oral, *Otodectes cynotis*, Otocariosis, Topical

## Abstract

**Background:**

The efficacy of fluralaner for the treatment of *Otodectes cynotis* infestations in dogs and cats was evaluated after oral (dogs) or topical administration (dogs and cats).

Twenty-four dogs and sixteen cats were experimentally infested with *O. cynotis* and randomly allocated to equal sized groups (*n* = 8/group). Dog groups were treated once, either orally with fluralaner at a minimum dose of 25 mg/kg body weight, topically with fluralaner at a dose of 25 mg/kg body weight or topically with saline solution (control). Cat groups were treated once, either topically with fluralaner at a dose of 40 mg/kg body weight or topically with saline solution. Ears of all animals were examined otoscopically for live visible mites and the amount of debris and cerumen before, and 14 and 28 days after treatment. Twenty-eight days after treatment, animals were sedated and both ears were flushed to obtain the total number of live mites per animal. The efficacy was calculated, based on the results of the ear flushing, by comparing mean live mite counts in the fluralaner treated groups versus the saline solution treated group.

**Results:**

A single topical treatment of cats with fluralaner reduced the mean mite counts by 100% (*P* < 0.001) at 28 days after treatment. Similarly, a single oral or topical treatment of dogs with fluralaner reduced the mean mite counts by 99.8% (*P* < 0.001) at 28 days after treatment. Cats treated topically with fluralaner had no mites visible during otoscopic examination at either 14 or 28 days after treatment. All dogs treated orally or topically with fluralaner had no mites visible during otoscopic examination at 28 days after treatment. At 14 days after treatment, only 1–2 mites were visible in three dogs (oral treatment: 2 dogs, topical treatment: 1 dog). All fluralaner-treated animals showed improvement in the amount of cerumen exudation compared with observations performed before treatment. No treatment related adverse events were observed in any dogs or cats enrolled in these studies.

**Conclusions:**

In this study, fluralaner administered topically to cats and orally or topically to dogs was highly effective against *Otodectes cynotis* mite infestations.

## Background

*Otodectes cynotis* (“ear mites”) are a common cause of otitis externa, particularly in cats but also in other animals, including dogs, ferrets, foxes and occasionally humans [1–3]. These mites are non-burrowing obligate parasites belonging to the family Psoroptidae that live mainly on the horizontal and vertical ear canal lining surfaces, but are also occasionally seen on the body (e.g. head, feet and tip of the tail) [4]. The life-cycle occurs entirely within the ear, includes four stages (egg, larvae, nymph, adult) and can be completed in about 3 weeks [5].

Ear mite infestation (“otocariosis”) is very contagious [6]; occurs in dogs and cats worldwide; and is commonly diagnosed during routine veterinary physical examinations. Up to 85% of otitis externa cases in cats and up to 50% of such cases in dogs are estimated to be caused by *O. cynotis* [7]. Puppies and kittens, particularly cats between 3–6 months of age [8], appear to be more commonly affected than older animals, possibly because of acquired immunity [6, 9]. The main route of infestation is from infested dams/queens to their puppies/kittens, but mites can also be spread by contaminated combs, brushes, bedding and other grooming accessories [10]. Ear mites primarily feed on desquamated epithelial cells and aural exudates but will occasionally pierce the ear lining and feed on tissue fluids [11]. Otitis externa in infested dogs and cats is characterized by vertical and horizontal ear canal erythema with a characteristic dark brown, ceruminous otic exudate. Papulocrustous lesions (miliary dermatitis) may be found on the head, feet and tip of the tail, if mites migrate out of the ear canal [12]. Clinical signs include marked pruritus, mild to severe dermatitis, frequent scratching of the ears, and head shaking. The intense pruritus may result in self-mutilation, bleeding and aural haematoma development [10]. Occasionally, the infestation leads to intense irritation and secondary bacterial infection, possibly resulting in purulent otitis externa [13].

Fluralaner is an isoxazoline ectoparasiticide, that provides immediate and persistent efficacy against ectoparasites including ticks and fleas on dogs and cats [14]. Oral fluralaner treatment is effective against generalized *Demodex* mite infestation on dogs [15] and oral and topical fluralaner treatment are effective against *Sarcoptes scabei* infestation on dogs [16], whereas there are no reports regarding efficacy against mites on cats. Fluralaner (Bravecto™) is commercially available as a chewable tablet for dogs or as a spot-on solution for cats. A spot-on solution is also licensed in some countries for use on dogs. These fluralaner formulations were evaluated for treatment of infestations with *O. cynotis* in dogs and cats.

## Methods

### Study set-up

Two studies, one in dogs and one in cats, were conducted in accordance with Good Clinical Practice (VICH guideline GL9, Good Clinical Practice, EMA, 2000). The study set-up was a parallel group, masked, randomized, and controlled efficacy design. Masking of the study personnel was assured through the separation of study functions, i.e. personnel conducting observations, animal care or performing mite examinations and counts after treatment were masked to treatment allocation.

### Animal details

Dogs included were mixed breed (mainly mongrels) and of both sexes (18 males, 6 females), between 1 and 9 years old and weighing between 8.8 and 24.2 kg on the day of treatment. Cats included were European mixed breed (short hair) and of both sexes (6 males, 10 females), between 10 weeks and 5 years old, and weighing between 2.3 and 4.7 kg on the day of treatment.

*Otodectes cynotis* infestations were established in all animals by harvesting mites by lavage from donor animals and transferring approximately 50 to 100 mites, depending on the intensity of infestation in donor animals, into each ear of the recipient animal. Donor animals were either naturally infested animals from the field or experimentally infested animals from the test site. Donor dogs were used for the dog study and donor cats were used for the cat study. Animals used in the study were experimentally infested within one month prior to study start. Before study inclusion, the presence of live *O. cynotis* mites in both ears was confirmed in each animal by otoscopic examination. None of the animals had been treated with fluralaner or any other product with an acaricidal/insecticidal effect for at least 8 weeks prior to treatment. In each study, the animals were blocked within sex by descending pre-treatment live mite and debris score, randomly allocated to one of three study groups in the dog study or to one of two study groups in the cat study, with groups consisting of eight animals each. All animals were healthy upon physical examination, apart from otic signs of ear mite infestation, prior to treatment.

For the duration of the study, dogs were housed individually in an indoor/outdoor run, and cats were housed in groups of 2–3 cats within their study group in an outdoor pen. Physical contact between animals (dogs) or animals of different groups (cats) was not possible. All animals were fed a species specific food once a day, according to the manufacturer’s recommendations and had free access to potable municipal water.

### Treatment

One group of dogs was treated once orally with fluralaner chewable tablets, at the minimum recommended dose of 25 mg/kg body weight. Doses were determined based on the individual body weights and the nominal content of fluralaner in the tablets. Dogs received a single whole tablet or a combination of tablets to achieve a dose as close as possible to the calculated target fluralaner dose without underdosing (dose range 25.4–32.7 mg/kg body weight). Chewable tablet(s) were administered 20 (±10) minutes after food had been offered by placing the tablet(s) in the back of the mouth over the tongue to initiate swallowing. No vomiting or regurgitation was observed in any treated dog. A second group of dogs was treated once topically with fluralaner at a dose of 25 mg/kg body weight. One group of cats was treated once topically with fluralaner at a dose of 40 mg/kg body weight. Cats and dogs in the control groups and dogs treated orally with fluralaner chewable tablets were treated once topically with saline solution, to maintain masking. The volume of saline solution was equivalent to the volume of fluralaner solution applied to the treated groups. Before topical administration of fluralaner or saline solution using a disposable 1 ml plastic syringe, the skin and hair of the designated administration site(s) were inspected visually to confirm absence of any abnormalities. For administration, the animal was held in a standing position, the animal’s hair was parted and the tip of the syringe was placed vertically on the skin and the fluralaner or saline solution administered directly to the skin. Dogs and cats were topically administered one or more spots according to the product label administration recommendations. No evidence of mis-dosing, such as spillage or run-off/drip-off, was observed in any treated animal.

### Assessment of mite infestation

An otoscopic examination of both ears from each animal was performed prior to treatment and at 14 and 28 days after treatment. At each otoscopic examination the number of visible live mites was counted in each ear, with the following clustering of results: 0 live mites; 1–4 live mites; 5–10 live mites; or > 10 live mites. For study inclusion, an animal was regarded as adequately infested if there were > 10 live mites in each ear. Additionally, at the same time points, the amount of debris/cerumen in each ear was recorded as no debris/cerumen, slight debris/cerumen, moderate debris/cerumen, or severe debris/cerumen.

Twenty eight days after treatment, animals were sedated and both ears were flushed to determine the number of live mites. The ear duct was filled with 5% aqueous solution of docusate sodium (Docusol®, 5% aqueous solution of docusate sodium, Kyron Laboratories) and slightly massaged to soften the ear duct content. The solution was removed from the ears and filtered through a 38 μm sieve. The ears were then flushed with lukewarm saline solution, which was poured through the same sieve. The ears were examined otoscopically and, if needed, the flushing process was repeated until the ear ducts were assessed as clean (no visible cerumen or mites). Sieve contents were rinsed with water and transferred to a Petri dish and all live mites (adults, larvae, nymphs) were counted under a stereo microscope.

### Animal health

General health observations were performed once daily throughout the complete study duration.

### Efficacy evaluation

Statistical analysis was performed using the software package SAS® (SAS Institute Inc., Cary, NC, USA, release 9.3 TS Level 1 M2), using the individual animal as experimental unit. The primary assessment variable in each study was the total number of live mites (sum of adults, nymphs and larvae from both ears) counted during ear flushing on Day 28 after treatment. The percentage of efficacy against *O. cynotis* mites was calculated using geometric means employing Abbott’s formula:

$$ \mathrm{Efficay}\kern0.5em \left(\%\right)=100\times \left({\mathrm{M}}_{\mathrm{C}}-{\mathrm{M}}_{\mathrm{T}}\right), $$

where M_C_ was the mean number of total live mite counts in the saline treated group, and M_T_ the mean number of total live mite counts in each fluralaner-treated group. Significant differences were assessed between the log-transformed [ln(x + 1)] counts of live *O. cynotis* mites in the fluralaner treated groups compared to the log-transformed [ln(x + 1)] counts of the saline treated group using a mixed analysis variance model including study group as a fixed effect and block as a random effect. The two-sided level of significance was set as *P* ≤ 0.05 (One-way ANOVA with a treatment effect).

## Results

No adverse events related to oral or topical fluralaner administration were observed in any cat or dog at any time during the studies.

All included dogs had an adequate mite infestation in both ears, except for one dog that had an otoscopic mite count of 5–10 mites in one ear. However, this dog had a severe debris/cerumen build up in both ears and > 10 live mites in the other ear and it was therefore judged as adequately infested for inclusion. From the included cats, 9 cats had an adequate mite infested in both ears, 5 cats had an otoscopic mite count of > 10 mites in one ear and between 1–10 mites in the other ear and two cats had an otoscopic mite count of 1–4 mites in both ears. Nevertheless, due to the presence of debris/cerumen, all cats were judged to be adequately infested and included into the study.

**Fig. 1:**
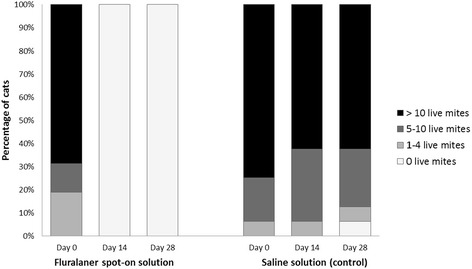
Cats with visible live mites observed during otoscopic examinations before and 14 and 28 days after treatment

**Fig. 2:**
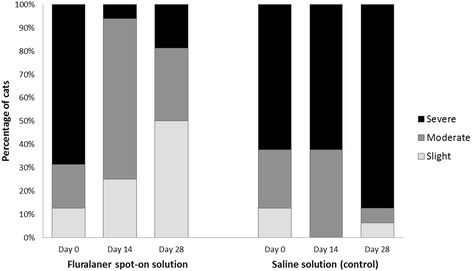
Cats with cerumen/debris observed during otoscopic examinations before and 14 and 28 days after treatment

In cats, after ear flushing 28 days after treatment, no mites were observed in any fluralaner treated animal, whereas a mean of 595.1 live mites was recorded in control cats, thus resulting in a significant (*P* < 0.001) mite reduction and efficacy of 100% (Table 1). Cats treated topically with fluralaner had no mites visible during otoscopic examination at either 14 or 28 days after treatment (Fig. 1). Fluralaner treated cats showed improvement in the amount of cerumen/debris by 28 days after treatment (Fig. 2).

**Table 1:** Mite counts and corresponding efficacy (%) of fluralaner administered once topically (cats and dogs) or orally (dogs) against infestations with *O. cynotis* at 28 days after treatment

	Range (*n*)	Mean (*n*)^a^	Efficacy (%)
Cats			
Topical treatment	0	0	100 (*P* < 0.0001^b^)
Negative control	6–1,843	197.5	na
Dogs			
Topical treatment	0–1	0.1	99.8 (*P* < 0.0001^c^)
Oral treatment	0–1	0.1	99.8 (*P* < 0.0001^d^)
Negative control	24–664	58.9	na

*Abbreviation*: *na* not applicable

^a^Geometric mean

^b^One-way ANOVA with a treatment effect (*F*_(2, 21)_ = 58.44)

^c^One-way ANOVA with a treatment effect (*F*_(2, 21)_ = 100.07)

^d^One-way ANOVA with a treatment effect (*F*_(2, 21)_ = 100.07)

**Fig. 3:**
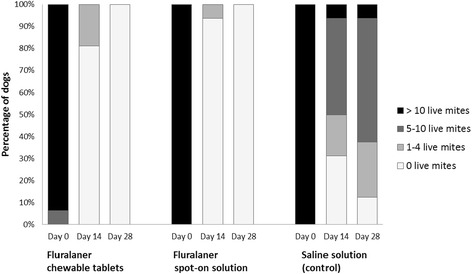
Dogs with visible live mites observed during otoscopic examinations before and 14 and 28 days after treatment

**Fig. 4:**
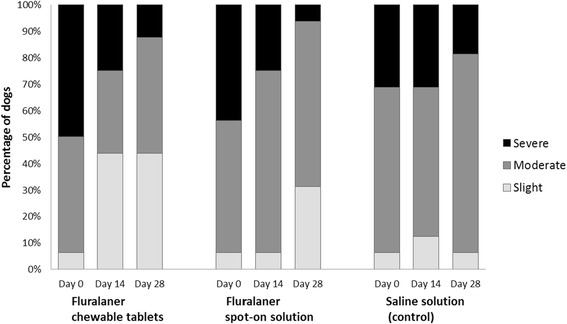
Dogs with cerumen/debris observed during otoscopic examinations before and 14 and 28 days after treatment

In dogs, after ear flushing 28 days after treatment, one animal in the topical fluralaner and one in the oral fluralaner treated group was found with one live adult mite in one ear, whereas a mean of 58.9 live mites was recorded in control dogs. The calculated efficacy was therefore 99.8% (statistically significant, *P* ≤ 0.001), for both, orally and topically fluralaner-treated dogs (Table 1). All dogs treated orally or topically with fluralaner had no mites visible during otoscopic examination at 28 days after treatment. At 14 days after treatment, only 1–2 mites were visible in three dogs (oral treatment: 1 dog with 1 mite, 1 dog with 2 mites; topical treatment: 1 dog with 1 mite), whereas all other dogs had no visible mites (Fig. 3). Improvement in the amount of cerumen/debris was observed in both orally and topically fluralaner-treated groups by 28 days after treatment (Fig. 4).

## Discussion

Topical fluralaner administration completely eliminated ear mites from infested cats, and both, oral and topical fluralaner administration, was 99.8% effective against ear mite infestations in dogs at 28 days after treatment. At 14 days after treatment, no mites were visible during otoscopic examination of any cat and only one mite was visible in one dog (topical treatment) or two mites in two dogs (oral treatment) were visible. This immediate mite decrease was observed together with improvement of otic clinical signs seen in both dogs and cats after fluralaner treatment. The study observation period was 28 days; however, the duration of efficacy of fluralaner against other ectoparasites is up to 12 weeks [14] and therefore it is assumed that efficacy and further improvement of otic signs will continue beyond this study period.

Fluralaner efficacy against cutaneous mites is likely associated with the presence of fluralaner in extracellular fluids found in the dermal tissues. Fluralaner (Bravecto™) is effective against other cutaneous mite infestations of dogs including generalized demodicosis [15] and sarcoptic mange [16]. This is the first study showing efficacy of fluralaner treatment against a mite infestation of cats.

Ear mite infestations are of greater clinical importance in cats than in dogs. Control cats showed a tenfold higher number of mites compared to control dogs at the end of the 28 day observation period, although all animals were initially experimentally infested with the same number of mites.

Existing commercially available products for the treatment of *Otodectes cynotis* infestation in dogs and cats either consist of one (selamectin) or a combination of two (imidacloprid and moxidectin) pharmaceutical ingredients. Efficacy over 4 weeks of these products is reported following either a single administration [9, 17] or two administrations one month apart [9, 13, 17, 18]. The efficacy results, assessed one month after treatment, are in line with the results shown here after a single treatment of dogs and cats with fluralaner.

The 28 day period in this study protocol between treatment of dogs and cats and the subsequent assessment of clinical signs was too short to allow complete resolution of ear mite associated presence of debris and cerumen. Fluralaner treatment provides systemic ectoparasiticide efficacy for up to 12 weeks against ticks and fleas on dogs and cats [14] and therefore fluralaner administration might provide sustained control of ear mite infestations in susceptible dogs and cats following treatment. Fluralaner could provide a convenient option to provide resolution of ear mite infestations in addition to its immediate and persistent activity against fleas, ticks and mange mites on treated dogs and cats.

## Conclusions

Topical administration of fluralaner was 100% effective for elimination of ear mites infestations in cats, and oral and topical fluralaner administration are 99.8% effective against ear mites in dogs 28 days after treatment. In both studies, fluralaner treatment resulted in an improvement of otic clinical signs during the 28 day observation period.
